# Hypofractionated Radiotherapy With Volumetric Modulated Arc Therapy Decreases Postoperative Complications in Prosthetic Breast Reconstructions: A Clinicopathologic Study

**DOI:** 10.3389/fonc.2020.577136

**Published:** 2020-11-17

**Authors:** Seung Yong Song, Jee Suk Chang, Kenneth L. Fan, Mi Jung Kim, Hsien Pin Chang, Dae Hyun Lew, Tai Suk Roh, Hyun Roh, Yong Bae Kim, Dong Won Lee

**Affiliations:** ^1^Department of Plastic & Reconstructive Surgery and Institute for Human Tissue Restoration, Yonsei University College of Medicine, Seoul, South Korea; ^2^Department of Radiation Oncology, Yonsei University College of Medicine, Seoul, South Korea; ^3^Department of Plastic and Reconstructive Surgery, MedStar Georgetown University Hospital, Washington DC, United States

**Keywords:** prostheses and implants, mammaplasty, dose hypofractionation, radiotherapy, intensity-modulated, postoperative complications

## Abstract

**Background:**

Emerging radiation technologies are expected to provide a positive impact on the reduction in postoperative complications in patients receiving prosthetic breast reconstruction. This study aimed to determine whether hypofractionated radiation therapy(RT) with volumetric modulated arc therapy(VMAT) is superior to conventional RT in the setting of prosthetic reconstruction.

**Methods:**

From retrospective data collections, postoperative complications were compared for all patients with mastectomy and staged prosthetic reconstruction without RT, with hypofractionation using 40 Gy in 15 fractions with VMAT (Hypo-VMAT) or conventional RT (50 Gy over 5 weeks). After harvesting subpectoral capsules from patients with informed consents, histologic analysis including immunohistochemistry and immunofluorescence for collagen type I, α-smooth muscle actin, CD34 and CD31 expression was performed.

**Results:**

A total of 288 reconstructions without RT, 55 reconstructions with Hypo-VMAT, and 29 reconstructions with conventional RT were examined. During average follow-up period of 34.8 months, rates of overall complications were 6.3% in the no-radiation group, 18.2% in Hypo-VMAT group and 44.8% in conventional-RT group with significant differences (no-RT vs Hypo-VMAT: *p*=0.006; Hypo-VMAT vs conventional-RT: *p*=0.012). Levels of myofibroblasts and tissue fibrosis were lower in the Hypo-VMAT group than in conventional-RT group (*p=*0.016 and *p=*0.040, respectively), while those of progenitor cells and microvessel density were higher in the Hypo-VMAT group than in conventional-RT group (*p*<0.001 and *p*<0.001, respectively).

**Conclusion:**

We demonstrated that hypofractionated RT with VMAT served to reduce radiation-related morbidities in prosthetic reconstruction from a clinicopathologic perspective, compared to conventional RT. It may offer a practical strategy to mitigate radiation-related complications in clinical settings.

## Introduction

Breast cancer treatment continues to undergo paradigmatic evolution in the field of oncologic and reconstructive surgery. Due to insurance reimbursements in respective localities, increased incidence of contralateral prophylactic mastectomy, and advances in surgical technique, rates of prosthetic based breast reconstruction are growing (1, 2). The indications for post-mastectomy radiation therapy(PMRT) have been expanded due to evidence of benefit local control and survival in early breast cancer with a positive axillary node as well as advanced and intermediate-risk breast cancer (3, 4). However, the combination of the PMRT and prosthetic based breast reconstruction significantly increases complication rates, putting the treating physicians in a challenging situation (5, 6).

Previously, breast reconstruction with a prosthetic device combined with irradiation had not been recommended due to high reconstruction failure rate and negative impact on patient satisfaction (5–7). Recent studies report that complications and aesthetic results of prosthetic reconstruction in the setting of PMRT have improved to acceptable levels compared to prior studies (8–11). Improved outcomes may be attributable to newer prostheses, staged prosthetic reconstruction, use of acellular dermal matrix and advancements in radiation therapy (RT). Furthermore, advances in radiation techniques such as prone positioning are expected to reduce complication rate by decreasing the maximum skin dose (12).

New advances in the use of hypofractionated radiation are gaining wide popularity due to patient convenience and reduced cost of care (13, 14). The oncologic rationale for hypofractionated treatment is based on the emerging evidence that breast cancer is more sensitive to fraction size than previously thought, implying that conventional fractionation with small fractions has no advantage (15). Multiple prospective randomized trials established the role of hypofractionated RT in whole breast irradiation and PMRT: local control was equal, and cosmetic results and complications were similar or better with hypofractionation compared with conventional fractionation (13, 16–18).

When considering radiation delivery, intensity-modulated radiotherapy (IMRT) represents the next evolution of conforming techniques. Prior, 3-dimensional conformal RT(3D-CRT) that is currently more popular in most countries, utilizes multiple uniform beams to spatially plan the distribution of radiation dosage. This technique relies on forward planning, whereby the treatment parameters (beams, shape, intensity, angle) are decided, and resultant dose distribution are evaluated. However, healthy tissues may not be completely excluded. IMRT incorporates nonuniform intensity of radiation beams by modulating each beam’s photon fluence (12, 19). Volumetric modulated arc therapy(VMAT) is a new type of IMRT, which continuously optimizes the radiation intensity as it rotates around the patient body. Computerized inverse planning allows radiation oncologists to select the desired dosage distribution for better dose homogeneity to the tumor, after which computerized models optimize treatment parameters to spare surrounding tissues (20).

In the process of adopting the newer radiation techniques, hypofractionated RT and VMAT for PMRT at our institution, we were able to observe a reduction in RT-related complications in patients receiving prosthetic reconstruction. Herein, we seek to compare clinical and histological outcomes between patients undergoing prosthetic breast reconstruction and hypofractionated RT with VMAT, conventional RT with 3D-CRT, and no irradiation.

## Materials and Methods

### Patient Selection

A retrospective review of all breast cancer patients undergoing mastectomy and immediate prosthetic reconstruction between July 2012 and April 2018 was performed. This study was only limited to patients (N=373) treated with a consistent technique of immediate two-staged expander/implant reconstruction using acellular dermal matrix by two experienced plastic surgeons (D.W.L and S.Y.S) to limit bias related to differing types of reconstruction and surgeon experience. Five experienced oncologic surgeons performed the mastectomies. Exclusion criteria included patients receiving RT before mastectomy, combined techniques such as prophylactic mastectomy, unfinished staged reconstruction, implant plus flap and delayed reconstructions.

A total of 373 patients were divided into two groups: one that received PMRT, and the other, categorized as the no-RT group, that did not. The former cohort was further stratified into receiving either conventional fractionation (50.4 Gy in 28 fractions) with 3D-CRT or hypofractionation schedule (40.05 Gy in 15 fractions) with VMAT. These groups were referred to as the conventional-RT or the Hypo-VMAT group, respectively. Patients who received other combinations of RT techniques were excluded from the study. Hypo-VMAT group was compared with no-RT group and conventional-RT group as controls. The study was approved by the Institutional Review Board of Severance Hospital, Yonsei University Health System (IRB No. 4-2019-0204).

### Reconstruction Protocol

Surgical techniques and reconstruction protocol were uniform for all patients. After the mastectomy, an expander was introduced under the pectoralis muscle and lower lateral portion of the implant was covered with an acellular dermal matrix sling. An expander was prefilled depending on the closing tension of mastectomy flap. Inflation of expander began two weeks after the first operation and recurred every two to three weeks. Prior to radiation therapy, the expander was deflated to reduce interference with RT (21). Immediately following completion of RT, re-expansion was performed every two to three weeks until the desired breast volume was reached. The second stage of reconstruction occurred at least 3 months after completion of RT. In all cases, expanders were removed and shaped implants were placed.

### Radiation Protocol

**Figure 1:**
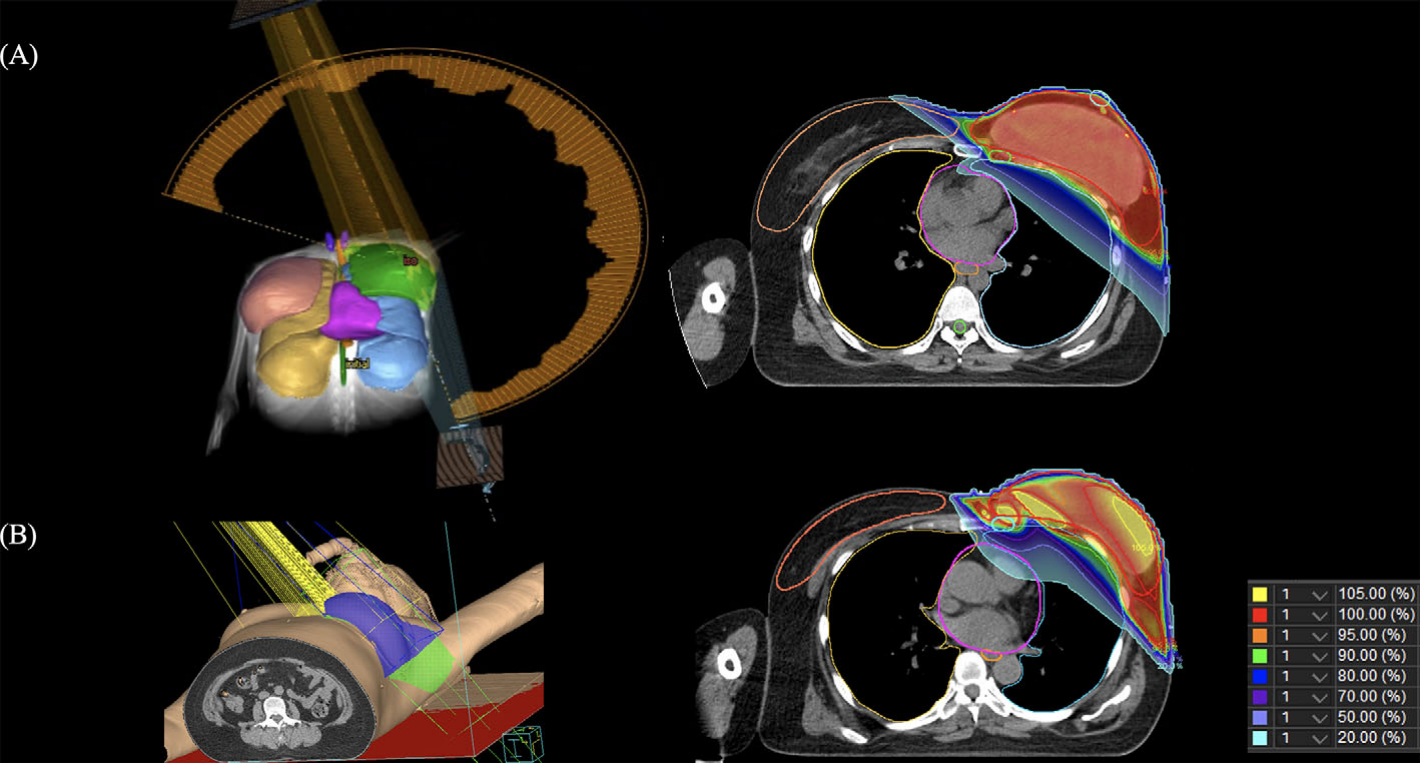
Images of the 3-dimensional rendering images and computed tomography slice showing the dose distribution. **(A)** volumetric modulated arc therapy, **(B)** 3-dimensional conformal radiotherapy.

PMRT was performed within 6 weeks after mastectomy or completion of the last cycle of adjuvant chemotherapy. Administered daily from Monday to Friday, targeted tissues included the ipsilateral chest wall, mastectomy scar and regional nodal basins including axillary nodes, internal mammary lymph nodes, and supraclavicular lymph nodes. RT techniques have been described in detail previously (22). Briefly, in the conventional-RT group, the chest wall was irradiated with 2 tangential photon beams and supraclavicular node with anterior photon beam with 50.4 Gy in 28 fractions of 1.8 Gy using the 3D-CRT technique. Bolus material was used in patients who have a high risk of skin recurrence to ensure that skin was covered adequately at the discretion of the physician. In the Hypo-VMAT group, the prescription dose was 40.05 Gy in 15 daily fractions of 2.67 Gy. Bolus material was used in patients who had a thin chest wall at physician’s discretion (**Figure 1**). Arc-based VMAT (Elekta Infinity Linac, Elekta, Crowley, UK) was used in all patients with hypofractionated regimen.

### Clinical Assessment

Patient information was collected from hospital electronic medical records including demographics and RT-related complications. Complications relevant to RT were categorized as capsular contracture, wound dehiscence with prosthesis exposure, peri-prosthetic infection, and cellulitis. Overall complication rate was defined as the rate of patients with at least one complication among those complications. Capsular contracture was defined as modified Baker classification grade III-IV using the modified Baker classification for reconstructed breasts (23). Complications that occurred prior to RT were excluded. Patient characteristics and complications were compared between groups. In addition, conditions that required major revisional surgery due to postoperative complications were examined. The major revisional surgery included implant change, explantation of the implant, and coverage with a latissimus dorsi flap.

### Histologic Analyses

Following patient consents (n=9 in each group), histologic samples were taken from the subpectoral capsules at the second stage breast reconstruction exchanging expander with a permanent implant. Harvested tissue samples were fixed with 10% formaldehyde, embedded in paraffin blocks and mounted on slides. The slides were analyzed according to immunohistochemistry and immunofluorescence protocol. Immunohistochemistry was performed to measure the amount of α-smooth muscle actin (α-SMA), collagen type I (col-1) and CD34 (progenitor cell marker), and microvessel density from the CD31-positive vessel count as previously described.^11^ Semi-quantitative analysis of the synthesis of α-SMA, col-1 and CD34 were executed using MetaMorph^®^ image analysis software (Universal Image Corporation, Buckinghamshire, UK) at high-power field (×400). To calculate the microvessel density, the CD31 positively stained vessels were counted at each low-power field (×100). Results were expressed as the average value of integrated optical density (IOD) or the average number of vessels in 45 different digital images (5 images per one patient; 9 patients per each group) and compared between groups.

Immunofluorescence was prepared with a standard protocol using the following primary and secondary antibodies: anti-col-1, anti-α-SMA, anti-CD34 and anti-CD31 (Abcam, Cambridge, MA, USA) and bovine anti-rabbit IgG-FITC and Texas Red-conjugated bovine anti-mouse IgG (Santa Cruz Biotechnology, Inc., Santa Cruz, CA, USA), respectively. Cells were viewed under a confocal microscope system (LSM700, Olympus, Center Valley, PA, USA).

### Statistical Analysis

All statistical analyses were conducted by using IBM SPSS Statistics for Windows, version 23.0 (IBM Corp., Armonk, N.Y., USA). To compare the mean of continuous variables (i.e., patient age, body mass index, breast volume, follow-up period, quantitative values of immunohistochemistry) among the three groups, one-way analysis of variance (ANOVA) test was used. The Chi-squared or Fisher’s exact test was used to assess any differences between categorical variables (i.e., histology, tumor stage, grade, receptor, adjuvant therapy, complication rate). Two-tailed values of *p*<0.05 were considered statistically significant.

## Results

### Patients Characteristics

Among a total of 373 patients who received mastectomy and immediate staged prosthetic reconstruction with shaped implants, 275 patients (288 reconstructions) were included in the no-RT group, 54 patients (55 reconstructions) in the Hypo-VMAT group and 29 patients (29 reconstructions) in the conventional-RT group. Sixteen patients with other combinations of RT protocol were excluded. The overall patient demographic and oncologic characteristics are presented in **Table 1**. No relevant difference in the distribution of the most characteristics between the groups was observed with the exception of age, pathologic stage, receptors (progesterone and triple negative) and neo-adjuvant chemotherapy. Patients without RT had an earlier stage compared to the other two groups, and distribution of the receptor and neo-adjuvant therapy showed a similar pattern of differences. However, there was no difference between Hypo-VMAT group and conventional-RT group in all demographic and oncologic characteristics. The average follow-up period in all patients was 34.8 ± 12.1 months.

**Table 1:** Patient demographic and oncologic characteristics.

Clinical parameter	No-RT group (n = 288)	Hypo-VMAT group (n = 55)	Conventional-RT group (n = 29)	*P*	*p^no-hypo^*	*p^no-cov^*	*p^hypo-cov^*
Age (year)	45.2 ± 8.9	41.7 ± 9.7	40.5 ± 10.2	0.003	0.033	0.028	>0.999
BMI (Kg/m^2^)	21.9 ± 3.2	22.0 ± 2.9	22.2 ± 3.3	0.891			
Breast volume (ml)*	323 ± 181	324 ± 146	317 ± 155	0.984			
Tumor histology				0.415			
IDC	231 (80%)	43 (78%)	26 (90%)				
Others	57 (20%)	12 (22%)	3 (10%)				
Final stage				<0.001	<0.001	<0.001	0.855
0	84 (29%)	10 (18%)	4 (14%)				
I	139 (48%)	8 (15%)	4 (14%)				
II	59 (20%)	21 (38%)	14 (48%)				
III	6 (2%)	16 (29%)	7 (24%)				
Grade				0.081			
I	55 (19%)	7 (12%)	3 (10%)				
II	126 (44%)	34 (62%)	14 (48%)				
III	34 (12%)	8 (15%)	6 (21%)				
Unknown	73 (25%)	6 (11%)	6 (21%)				
Receptors							
Estrogen (+)	226 (78%)	41 (75%)	20 (69%)	0.450			
Progesterone (+)	178 (62%)	21 (38%)	11 (38%)	0.001	0.001	0.013	0.982
HER-2 (+)	75 (26%)	12 (21%)	6 (21%)	0.687			
Triple negative	14 (5%)	7 (13%)	6 (21%)	0.002	0.057	0.005	0.357
Adjuvant therapy							
Neo-adj Chemo	5 (2%)	33 (60%)	14 (48%)	<0.001	<0.001	<0.001	0.303
Hormonal therapy	205 (71%)	40 (73%)	20 (69%)	0.936			
Chemotherapy	99 (34%)	19 (35%)	13 (45%)	0.529			
Follow-up (months)	34.8 ± 13.0	34.4 ± 8.2	35.4 ± 10.8	0.924			

All values are described as mean value ± standard deviation or number (percentage).

*Breast volume was three-dimensional surface imaging (Axis Three, AX3 Technologies, Miami, FA) (24).

RT, radiotherapy; HER-2, human epidermal growth factor receptor 2; N/A, not applicable.

p^no-hypo^, between no-RT group and Hypo-VMAT group; p^no-cov^, between no-RT group and conventional-RT group; p^hypo-cov^, between Hypo-VMAT group and conventional-RT group.

### Postoperative Complications

The rate of patients with at least one complication was lower for the no-RT group (6.3%) compared with the Hypo-VMAT group (18.2%) and the conventional-RT group (44.8%) (*p*=0.006 and *p*<0.001, respectively), and there was a significant difference between Hypo-VMAT group and conventional-RT group (*p*=0.012). Each type of complication was compared between three groups as follows: capsular contracture was present in 3.8% of no-RT group, 10.9% of Hypo-VMAT group, and 27.6% of conventional-RT group (*p*<0.001); wound dehiscence with prothesis exposure was present in 0.3, 9.1, and 24.1%, respectively (*p*<0.001); peri-prosthetic infection was present in 1.4, 3.6, and 10.3%, respectively (*p*=0.015); cellulitis was present in 0.7, 3.6, and 3.4%, respectively (*p*=0.106). The proportions of each complication were significantly different among the three groups, although there was no significant difference between Hypo-VMAT group and conventional-RT group (**Table 2**). Major revisional surgery was performed with the following frequencies: 1.0% of the no-RT group, 5.5% of Hypo-VMAT group, and 20.7% of conventional-RT group (*p*<0.001).

**Table 2:** Postoperative complications rate.

Complication type	No-RT group (n = 288)	Hypo-VMAT group (n = 55)	Conventional-RT group (n = 29)	*p*	*p^no-hypo^*	*p^no-cov^*	*p^hypo-cov^*
Capsular contracture	11 (3.8%)	6 (10.9%)	8 (27.6%)	<0.001	0.039	<0.001	0.067
Wound dehiscence	1 (0.3%)	5 (9.1%)	7 (24.1%)	<0.001	<0.001	<0.001	0.098
Peri-prosthetic infection	4 (1.4%)	2 (3.6%)	3 (10.3%)	0.015	0.247	0.019	0.335
Cellulitis	2 (0.7%)	2 (3.6%)	1 (3.4%)	0.106	0.122	0.251	>0.999
Overall complications	18 (6.3%)	10 (18.2%)	13 (44.8%)	<0.001	0.006	<0.001	0.012
Major revisional surgery	3 (1.0%)	3 (5.5%)	5 (20.7%)	<0.001	0.055	0.001	0.058

All values are described as number (percentage).

Overall complications rate, the rate of patients with at least one complication among radiation-related complications such as capsular contracture, wound dehiscence, peri-prosthetic infection, and cellulitis.

RT, radiotherapy; p^no-hypo^, between no-RT group and Hypo-VMAT group; p^no-cov^, between no-RT group and conventional-RT group; p^hypo-cov^, between Hypo-VMAT group and conventional-RT group.

### Myofibroblast and Tissue Fibrosis

**Figure 2:**
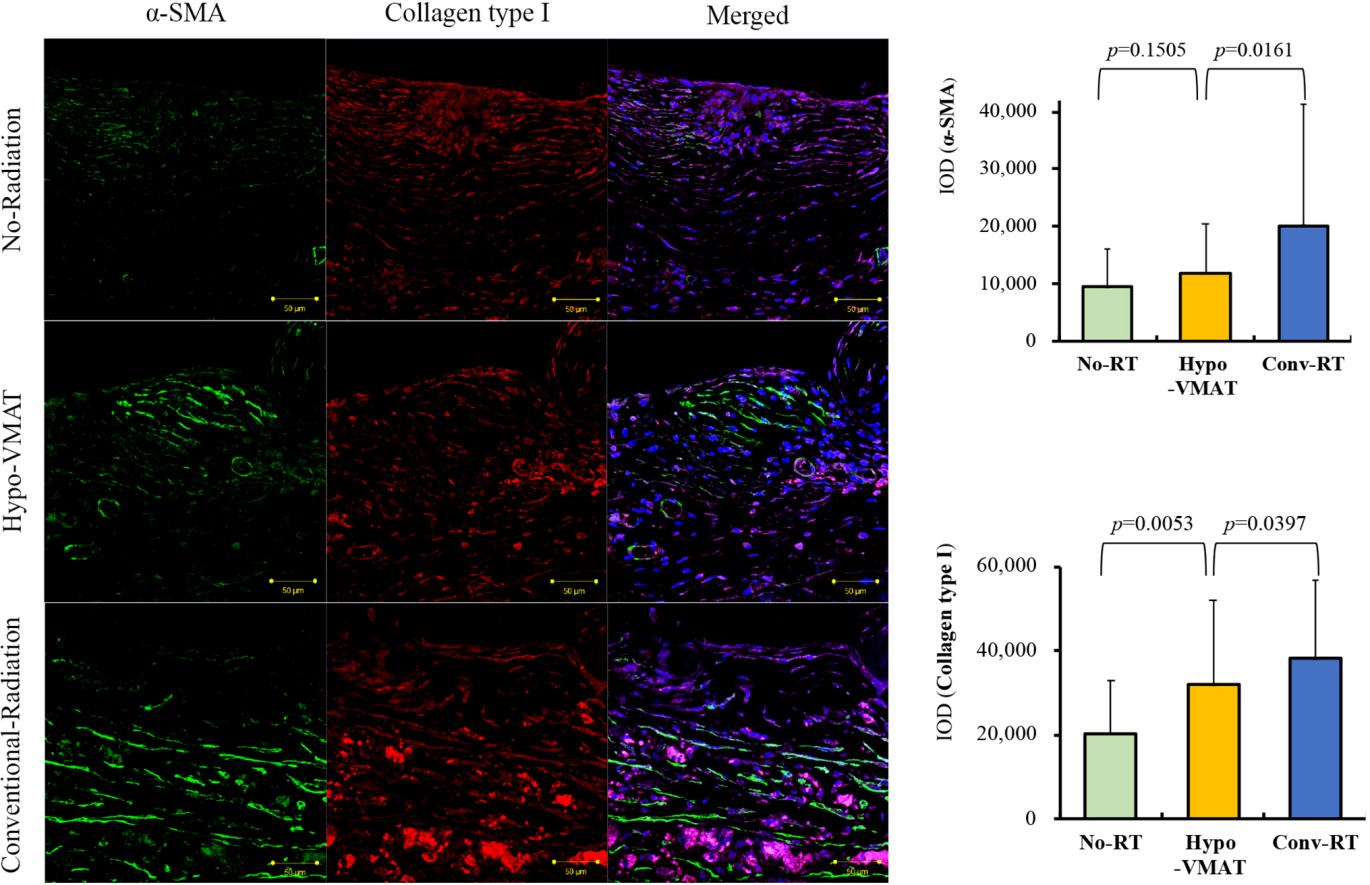
Expression of collagen type I and α-SMA. Immunofluorescence of collagen type I and α-SMA and semi-quantitative analysis with immunohistochemistry of collagen type I and α-SMA are shown. The expressions of collagen type I and α-SMA in Hypo-VMAT group are higher than those of no-radiation group, and lower than those of conventional radiation group.

Myofibroblast plays a crucial role in capsular contracture that is the most common complication in prosthetic breast reconstruction. We examined the amount of α-SMA expression using the value of IOD for immunohistochemically stained specimens to assess the levels of myofibroblasts (11, 25). A semi-quantitative analysis demonstrated that α-SMA expression was significantly different between the three groups (*p*<0.001) and significantly increased in the conventional-RT group (20,164 ± 21,056 IOD) compared with the no-RT group (9,504 ± 6,526 IOD; vs conventional-RT: *p*=0.002) and the Hypo-VMAT group (11,841 ± 8,615 IOD; vs conventional-RT: *p*=0.016). However, there was no statistical difference between the no-RT group and the Hypo-VMAT group (*p*=0.151) (**Figure 2**). These findings are congruent with clinical results, suggesting hypofractionated doses and VMAT delivery has a preventive effect on capsular contracture over conventional-RT technology. Next, we also measured the amount of col-1 expression in the same manner to access the degree of tissue fibrosis, which is relevant to capsular contracture clinically. Tissue fibrosis is attributed to excessive accumulation of collagen and other extracellular matrix components (26). The amount of col-1 expression was significantly different between the three groups (*p*<0.001). The conventional-RT group (38,077 ± 18,611 IOD) had a higher expression of col-1 than the no-RT group (20,379 ± 12,637 IOD; vs conventional-RT: *p*<0.001) and the Hypo-VMAT group (31,872 ± 20,045 IOD; vs conventional-RT: *p*=0.040). The difference between the no-RT group and Hypo-VMAT group was also statistically significant (*p*=0.005) (**Figure 2**). These findings indicate that radiation-induced fibrosis in the capsules of the conventional-RT group stands out above that of the Hypo-VMAT group.

### CD34-Positive Progenitor Cells

**Figure 3:**
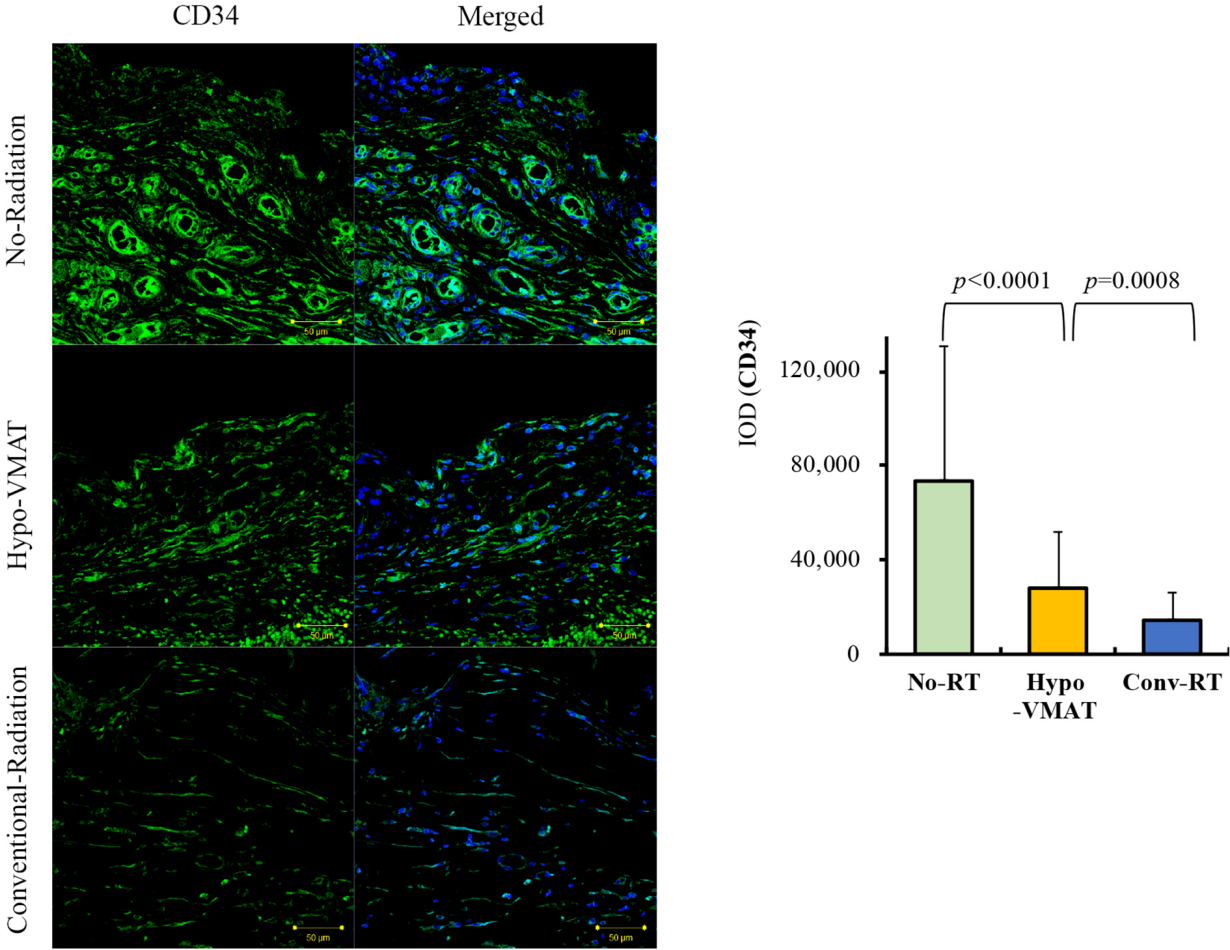
Expression of CD34. Immunofluorescence of CD34 in the three groups and semi-quantitative analysis with immunohistochemistry of CD34 are shown. The expression of CD34 in Hypo-VMAT group is lower than that of no-radiation group, and higher than that of conventional radiation group.

CD34 is regarded as a general marker of progenitor cells (27). We compared the level of CD34 expression between groups to evaluate how many progenitor cells remain in each capsular tissue. CD34 expression in the Hypo-VMAT group (28,206 ± 23,722 IOD) was significantly higher than the conventional-RT group (14,541 ± 11,254 IOD; vs Hypo-VMAT: *p*=0.001), but was significantly lower than the no-RT group (73,798 ± 56,891 IOD; vs Hypo-VMAT: *p*<0.001) (**Figure 3**). These findings suggest that hypofractionated doses and VMAT delivery is more likely to preserve progenitor cells than conventional technology.

### Microvessel Density

**Figure 4:**
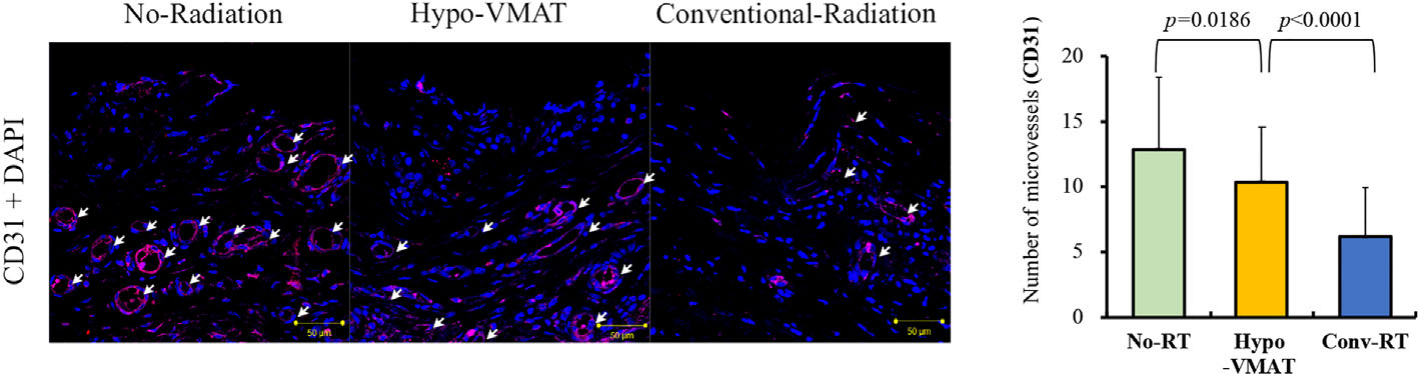
Microvessel density from the CD31-positive vessel count. Microvessel expression by immunofluorescence of CD31 and microvessel density with immunohistochemistry of CD34 are shown. The Microvessel density in Hypo-VMAT group is lower than that of no-radiation group, and higher than that of conventional radiation group. *White arrow*, microvessel.

The averages of CD31-positive vessels in samples from the no-RT group, the Hypo-VMAT group, and the conventional-RT group were 12.91 ± 5.54, 10.44 ± 4.12, and 6.18 ± 3.76, respectively. The difference between the Hypo-VMAT and conventional-RT group was significant (*p*<0.001), suggesting that microvascular depletion was substantial in the conventional-RT group rather than in the Hypo-VMAT group (**Figure 4**).

## Discussion

The combination of prosthetic reconstruction and PMRT is known to be vulnerable to postoperative complications and reconstruction failure. Accordingly, reconstructive surgeons reflexively select autologous over prosthetic reconstruction in patients who are potentially PMRT candidates. However, with multidisciplinary coordination between the oncologic surgeon, reconstructive surgeon, and radiation oncologists, satisfactory outcomes after prosthetic reconstruction are possible. Delayed-immediate breast reconstruction described by Kronowitz (28) is one such protocol. Furthermore, emerging evidence demonstrates the possibility to overcome RT-related complication in prosthetic reconstruction through strategies such as optimization of reconstruction protocol (29–31), acellular dermal matrix use (11, 25–32), and prepectoral implantation (33). Reconstructive surgeons are continually striving to reduce RT-related complications and reconstruction failure, resulting in the development of operative techniques and a multi-disciplinary approach. Nevertheless, prosthetic reconstruction in the setting of PMRT is still challenging.

Simply put, the adverse effects of radiation results from collateral damage in radiation delivery. Minimizing injury to healthy tissues has remained an end-goal, but has been elusive. A recent study by Muresan et al. found that higher degree of hot spots in breast skin was associated with RT-related complications; they suggested prone positioning RT as a means to decrease the maximum skin dose (12). In our previous study, we showed the results of dosimetric analysis: conventional fractionation regimen was a major determinant of hot spot level within the target volume compared to hypofractionated regimen (22). Our current data indicates that patients who receive hypofractionated radiation with VMAT are less likely to develop RT-related complications than those receiving conventional RT. These results suggest that new radiation technology using hypofractionated radiation and VMAT reduces radiation delivery to the surrounding normal tissues compared to conventional RT. Although the Hypo-VMAT group was superior to the conventional-RT group, there was a clear difference between the Hypo-VMAT group and the no-RT group. Even with improved radiation delivery, collateral damage is not completely eliminated. We sought to elucidate the degree of efficacy of the new radiation technology between two controls, no-RT group and conventional-RT group.

These clinical findings were in accordance with the histologic results. The pathologic effects in the irradiated tissue are thought to result from the loss of stem and progenitor cells (26, 34). Therefore, well-preserved progenitor cells imply that normal tissues are spared from collateral damage. CD34, a progenitor cell marker, is expressed not only by mesenchymal stromal cells but by a multitude of other progenitor cells (35–38). The subpectoral capsule and around tissues theoretically include several progenitors such as mesenchymal stromal cells, muscle satellite cells, and vascular endothelial progenitors. The CD34 positive cells in this study are postulated to be such progenitor cells surviving after breast reconstruction with or without RT. Progenitor cells play a pivotal role to regenerate injured tissues, enhance wound healing, and prevent pathologic fibrosis. Given that these regenerative mechanism defective, capsular contracture and wound dehiscence may occur at higher rates. Furthermore, one of the main histological changes associated with radiation injury is vascular endothelial damage, which causes chronic microangiopathy followed by a decrease in tissue perfusion (34). Previous animal studies showed hyperemia in the short term and microvasculature depletion in the long term (39–41). Our finding in the conventional-RT group was in line with previous results, and microvessel density in the Hypo-VMAT group was different from that in the conventional-RT group, suggesting the new radiation technology exhibited less affect to the microvasculature.

As interest in hypofractionated radiation grows, studies addressing hypofractionated PMRT have been reported recently. A few researchers investigated the reconstructive outcomes of hypofractionated PMRT, but they did not prove the superiority of hypofractionated PMRT over the conventional method (15, 20, 42). Our prior work reported that reconstructive complication rate was lower in patients with hypofractionated RT than in patients with conventional RT (14.3 vs 38.5%: *p*=0.017) (22). This current study, an evolution of our previous work, reveals overall complications rates were 18.2% when hypofractionated RT was combined with VMAT, compared to 44.8% in conventional-RT group. The higher complication rate of conventional RT in this study compared to our previous one may be due to more extended median follow-up period (32.5 vs 36.1 months) and inconsistency of enrolled patients, although some are partially overlapped. This is the first study, as far as we are aware, in which histologic analysis is performed in patients who received hypofractionated RT for breast cancer. Nevertheless, the results of this study have limitations of the design as a retrospective study in small patient series not randomly assigned to groups. We have started a prospective multi-institutional study (NCT 03523078). It would be expected to provide a high level of evidence for patient-reported outcomes in patients treated with breast reconstruction and different fractionation regimens.

Prosthetic reconstruction in the setting of PMRT is a challenging situation which reconstructive surgeons would inevitably encounter. Because they cannot completely avoid it, there is a compelling need to find ways to overcome it. Our findings provide a strategy to mitigate radiation-related morbidities by newer radiation technologies that are not universally popular, demonstrating that hypofractionated breast irradiation with VMAT serves to reduce postoperative complications as well as the collateral damage to normal tissues with histologic evidence. Furthermore, to achieve optimal outcomes in breast reconstruction, especially in irradiated patients, the importance of a multidisciplinary approach between surgeons and physicians cannot be emphasized enough.

## Data Availability Statement

The original contributions presented in the study are included in the article/supplementary materials, further inquiries can be directed to the corresponding authors.

## Ethics Statement

The studies involving human participants were reviewed and approved by the Institutional Review Board of Severance Hospital, Yonsei University Health System. The patients/participants provided their written informed consent to participate in this study.

## Author Contributions

SS, JC, YK, and DWL drafted the manuscript and worked on the conception, design, and interpretation of the data. JC, MK, HC, HR, and DWL performed the data analysis. KF, DHL, TR, and DWL reviewed the data analysis and study conclusions. All authors contributed to the article and approved the submitted version.

## Funding

This study was supported by the Basic Science Research Program through the National Research Foundation of Korea (NRF) funded by the Ministry of Education (Grant number: 2018R1D1A1B07049448) and the Research Grant of the Korean Foundation for Cancer Research (Grant number: 2017-B-3).

## Conflict of Interest

The authors declare that the research was conducted in the absence of any commercial or financial relationships that could be construed as a potential conflict of interest.
